# The emerging role of group 3 innate lymphoid cells in the neonate: interaction with the maternal and neonatal microbiome

**DOI:** 10.1093/oxfimm/iqab009

**Published:** 2021-05-12

**Authors:** Julie Mirpuri

**Affiliations:** Department of Pediatrics, Division of Neonatal-Perinatal Medicine, UT Southwestern Medical Center, 5323 Harry Hines Blvd, Suite F3.302, Dallas, TX 75390-9063, USA

**Keywords:** lc3, neonatal, microbiome, innate lymphoid cells, maternal

## Abstract

Innate lymphoid cells (ILCs) are critical for host defense and are notably important in the context of the newborn when adaptive immunity is immature. There is an increasing evidence that development and function of group 3 ILCs (ILC3) can be modulated by the maternal and neonatal microbiome and is involved in neonatal disease pathogenesis. In this review, we explore the evidence that supports a critical role for ILC3 in resistance to infection and disease pathogenesis in the newborn, with a focus on microbial factors that modulate ILC3 function. We then briefly explore opportunities for research that are focused on the fetus and newborn.

## INTRODUCTION

At birth, host defense is critically dependent on innate immunity. The adaptive immune system only matures after birth with exposure to antigens, as demonstrated by the nonexistent or limited the presence of secondary lymphoid tissues in the newborn [1]. In contrast, innate immunity is tasked with developing and maturing in the fetus with minimal exposure to *ex utero* elements and necessity for tolerance to the maternal environment. Innate immune cells are also important for regulating maturation of adaptive immunity after birth. Understanding how the *in utero* environment modulates development of innate immunity has implications for neonatal and infant outcomes.

In particular, type 3 innate lymphoid cells (ILC3) are emerging as critical for the regulation of host defense, tissue repair and immune homeostasis during the perinatal period. Recent evidence also supports a role for the maternal and neonatal microbiome in modulation of ILC3 function *in utero* and shortly after birth. Of note, in human and murine studies, ILC3 have been implicated as a potential mediator in the pathogenesis of preterm birth [2], necrotizing enterocolitis (NEC) [3,4], late onset sepsis [5, 6], pneumonia [7] and bronchopulmonary dysplasia (BPD) [8] in preterm infants and neonates. Dissecting the relationship between the microbiome and development of ILC3 during the fetal and neonatal period has important clinical implications for development of therapeutics for prevention and treatment of these conditions, which carry a significant clinical burden [9].

In this review, we briefly describe the development, repertoire and function of ILCs with a focus on ILC3 during the neonatal period. We then examine recent evidence that support an association between the maternal and neonatal microbiome with ILC3 development and function. Finally, we discuss human and murine studies that explore the role of ILC3 in clinically relevant diseases in neonates.

### ILC: development and function

Cells of the innate immune system include neutrophils, macrophages, natural killer (NK) cells, dendritic cells and ILCs. ILCs were first described in 2008 and over the last 13 years groundbreaking work on ILC have allowed them to be defined and explored, elucidating to their critical role in host defense, metabolic and immune homeostasis and even the pathogenesis of cancer, metabolic disease, inflammation and allergy.

**Figure 1:**
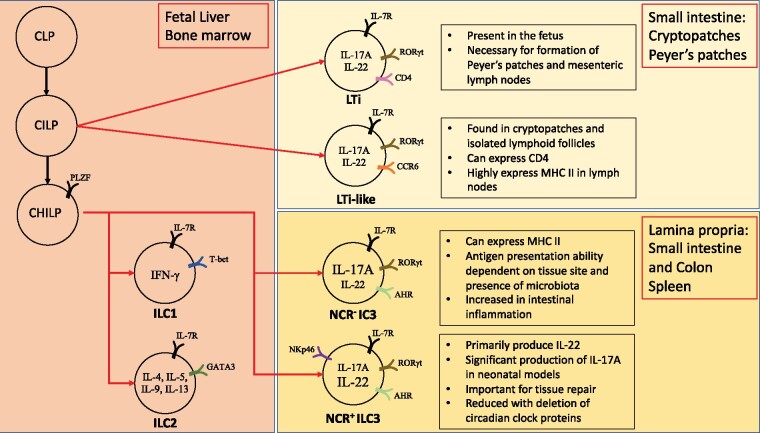
Development of Innate Lymphoid Cells

ILCs are tissue resident immune cells and are divided into three distinct groups based on the expression of transcription factors required for maturation. All ILCs and adaptive lymphoid cells are derived from a common lymphoid progenitor (CLP) present in the fetal liver and bone marrow (Fig. 1). Stimulation of CLPs by IL-7 results in commitment to the ILC lineages, and this common progenitor is known as common helper ILC progenitor (CHILP) [10]. CHILP expresses the transcriptional regulator inhibitor of DNA binding 2, known as Id2, and further differentiates into specific ILC lineages, with their nomenclature based on the transcription factor present (Table 1). Id2 deficiency results in failure of development of all ILCs.

**Table 1:** Characterization of Innate Lymphoid Cell subtypes

	Transcription factor	Cytokines produced	Predominant niche	Adaptive counterpart	Subtypes
Innate Lymphoid Cell (ILC)1	T-bet	IFN-γ	Multiple sites in blood and tissues	Th1 CD4+ T-cells	NK cells (Eomes^+^) are distinct ILC1
Innate Lymphoid Cell (ILC)2	GATA-3	IL-4 IL-5 IL-9 IL-13	Adipose tissue Lung	Th2 CD4+ T-cells	Natural and inflammatory subsets
Innate Lymphoid Cell (ILC)3	RORγt	IL-17A IL-22 GM-CSF	Intestine Skin	Th17 Th22	Very heterogenous LTi: critical for secondary lymphoid cell development NCR^+^ ILC3: primarily produce IL-22 (In neonatal mice, significant IL-17A production.) NCR^-^ ILC3: primarily produce IL-17A

Group 1 ILCs (ILC1) express the transcription factor T-bet and are found in multiple sites including blood and tissues in humans and mice. ILC1 produce IFN-γ and include NK cells. An increase in IFN-γ secreting ILC1 is associated with the pathogenesis of inflammatory bowel disease [11] and rheumatoid arthritis [12, 13]. Group 2 ILCs (ILC2) express the transcription factor GATA-3 and produce the cytokines IL-4, IL-5. IL-9 and IL-13. They are primarily found in adipose tissue and in the lung. Increased activation of ILC2 is implicated to play a significant role in allergic diseases [14, 15] and there is an expansion of intradermal ILC2 in atopic dermatitis [16, 17]. Group 3 ILCs (ILC3) express the transcription factor RORγt (Retinoid-related orphan receptor γt) and are primarily found in the intestine and skin. ILC3 primarily produce the cytokines IL-17A and IL-22, but they can also produce GM-CSF. IL-17A producing ILC3 have been implicated in inflammation while IL-22 producing ILC3 are critical for tissue repair and homeostasis [18]. Changes in the proportion of IL-17A and IL-22 producing ILC3 are associated with a range of pathologies in adults and neonates. An increase in IL-17A ILC3 is associated with the development of inflammatory bowel disease [19], multiple sclerosis [20] and NEC [3, 4], while a decrease in this subset is associated with the development of sepsis [5].

### Heterogeneity of ILC3: implications for the fetus and neonate

ILC3 are extremely heterogenous, with multiple subsets defined by expression of the natural cytotoxic receptor (NCR) NKp46 in mice and NKp44 in humans. Lymphoid tissue inducer (LTi) cells are a unique subtype of ILC3 that are NCR^−^ and CD4^+^ and critical for normal immune development during fetal life (Fig. 1). LTis are derived from an intermediate prior to CHILP, known as the common innate lymphoid progenitor (CILP), by the binding of Id2 to the precursor CLP. In the fetus, mice that lack Id2 are deficient in LTi’s and subsequently do not develop lymph nodes or Peyer’s patches [21]. Although commitment to the ILC1 and ILC2 lineage appears to occur in the bone marrow [10, 22], there is an evidence to support that final ILC3-lineage commitment occurs in the tissues, including the intestinal lamina propria, as ILC3 precursors have the capacity to migrate from the bone marrow [23–25]. This has significant biological implications in that exposure of fetal and neonatal tissues to differential environmental and microbial triggers can alter ILC3 lineage commitment. Indeed, it has been demonstrated that mature ILC3 lineage commitment can be influenced before and after birth by microbial colonization and the presence of dietary aryl-hydrocarbon receptor (AHR) ligands [26–28].

The presence, function, and cytokine production of ILC3 subtypes also vary across different tissues. For example, while ILC3 have been identified in the small intestine and spleen, the ability of ILC3 to present antigen to CD4^+^ T-cells is decreased in the small intestine. Germ-free mice have enhanced major histocompatibility complex II (MHC II) in the small intestine demonstrating that the microbiota drives antigen presentation by ILC3 [29]. This decrease in antigen presentation by ILC3 in the small intestine is driven by increased IL-23 production, which may be due to increased tissue IL-23 production by dendritic cells that are stimulated by microbial products [30]. A subtype of LTi ILC3 that produces both IL-17 and IL-22 (CCR6^+^ ILC3) is also only found specifically in cryptopatches and isolated lymphoid follicles in the small intestine. This ILC3 subtype programs development of specific dendritic cells (CIA-DCs) which produce IL-22 binding protein that blocks IL-22 and can regulate lipid absorption in the intestine [31].

Even within tissue sites, the presence and cellularity of subtypes of ILC3 are regulated. For example, T-bet deficient mice have increased numbers of NCR^−^ ILC3 in the colon [32]. Bacterial colonization and T-cell deficiency decrease the CCR6^+^ ILC3 subset in the mesenteric lymph nodes [33]. Within the intestine, there is a plasticity in ILC3 subtypes depending on the local cytokine and metabolite environment. ILC1 can differentiate into ILC3 in the presence of IL-1β and IL-23 [34]. ILC3 can also upregulate T-bet, express IFNγ and repress IL-17 and IL-22 secretion when exposed to IL-12, IL-18 and/or IL-1β [34]. Retinoic acid, a Vitamin A metabolite, enhances ILC3 homing from gut associated lymphoid tissue into the intestinal lamina propria and as a result, vitamin A deficiency decreases the total ILC3 in the gut [35]. Non-LTi ILC3 also express the AHR which is critical for the postnatal maintenance and production of IL-22 by ILC3 [18, 19]. AHR regulation of ILC3 is limited to postnatal ILC3, LTi ILC3 are not reduced in AHR deficient mice. AHR ligands include metabolites produced from microbial metabolism of the amino acid tryptophan and cruciferous vegetables found in the diet, as well as bilirubin, biliverdin, arachidonic acid derivatives and SCFA [36, 37].

ILC3 also utilize the circadian clock for homeostasis and interaction with the host microbiome. ILC3 express multiple circadian genes in a diurnal pattern and deletion of the clock proteins BMAL and REV-ERBα in mice results in reduction of NCR^+^ ILC3 in the intestine but with increased IL-17A and IL-22 production [38, 39]. In REV-ERBα deficient mice, NCR^−^ ILC3 subsets were at normal levels; however, the IL-17A secretion from these cells significantly increased [38]. Total ILC3 increased and cytokine production diminished in BMAL and REV-ERBα mice with microbiota depletion [39]. These data are intriguing and suggest that light, diet and the microbiome may be intricately involved with the regulation of ILC3 development, function and cytokine production. The significance of this in the context of the fetus and neonate remains to be explored.

## Differences between ILC3 in humans and mice

In humans, all the mature ILC subsets have been described, but there are still several deficiencies in our understanding of the intermediate cells involved in differentiation and their role in disease prevention and pathogenesis. ILCs in humans are derived from hematopoietic progenitor cells and differentiate into CHILP, similar to mice [40]. It is unknown if human ILC3 require Tbet and RORγt for their development. ILC3 in humans are defined by the presence of NKp44 as NCR^+^ or NCR^−^ and, similar to mice, produce the cytokines IL-17A and IL-22, as well as GM-CSF. In both humans and mice, IL-1β and IL-23 can differentiate NCR^−^ ILC3 into NCR^+^ ILC3s *in vitro* and differentiate ILC3 into CD127^+^ ILC1 on exposure to IL-12 [11]. A comparison of ILC3 in humans and mice is detailed in Table 2.

**Table 2:** Comparison of group 3 Innate Lymphoid Cells in humans and mice

	Human	Mouse
Sites present	Placenta Intestine Lung Skin	Placenta Intestine Lung
Subtypes	NCR designation based on presence of NKp44	NCR designation based on NKp46
Plasticity	ILC3 differentiates to ILC1 in presence of IL-2 and IL-12 in vitro [67], reversed by RA and IL-23 [68]	ILC3 can differentiate into ILC1 NKp46 ILC3 expression in gut is reversible [69]
Fetal development	LTi: mesenteric lymph nodes from 8 weeks gestation	CLP: fetal liver at E9.5-E14 LTi: fetal intestine from E12.5 Dependent on AHR and maternal retinoic acid
Cytokines produces	IL-17A IL-22 GM-CSF	IL-17A IL-22 GM-CSF
Post-natal development	Intestine: decreases with age	Dependent on AHR ligands and retinoic acid
Express TLRs	Yes, by transcript TLR 1, 2, 5, 6, 7 and 9	Not found
Association with pathology	Preterm birth, NEC	Sepsis, NEC, pneumonia, BPD

Timings of development of ILC3 have been well-described in mice. CLPs migrate to the fetal liver between embryological age E9.5 and E14. These cells differentiate into LTi and ILC3 precursors and can be found in the fetal intestine and in Peyer’s patches from E12.5 until birth [41]. In humans, ILC3 are also present early in fetal life since LTi cells are present in the fetal mesentery and lymph nodes as early as 8–9 weeks gestation (first trimester) [42]. The timing of emergence of other ILC3 populations in tissues is still unclear. From postmortem samples, the presence of ILC3s have been described in the human intestine as early as 2 years old, and up to 70 years old, with populations decreasing with age in the intestine [43].

Interestingly, human ILC3 express toll-like receptors (TLRs) [44], while murine ILC3 do not, which suggest that human ILC3 may be modulated directly by bacteria or their products. TLR 1, 2, 5, 6, 7 and 9 mRNA transcripts have been found in human ILC3 [44]. These known differences in ILC3 between humans and mice highlight the need for clinical and translational studies on ILC3 function and heterogeneity in preterm infants and neonates, which has been under investigated. This will enhance our understanding of the importance of ILC3 subtypes in population specific disease pathogenesis and direct the focus for mechanistic studies using murine models.

### Role of the maternal and neonatal microbiome on ILC3 development and function

The role of the maternal microbiome in ILC3 development was elegantly studied by Gomez de Aguero *et al.* [26] They demonstrated that transient colonization of the pregnant germ-free dam was sufficient to expand ILC3 in offspring. In the study, germ-free mice were colonized with an *Escherichia coli* strain that cannot persist in the intestine, resulting in only transient colonization and obviating the effect of exposure to bacteria during delivery. The offspring showed a persistent expansion of ILC3, seen up to 8 weeks of life, akin to adulthood. As live bacteria were not found in the placenta in treated mice, the effects were likely from exposure of the fetus to microbial metabolites (Ahr ligands), bound to maternal antibodies. Maternal retinoids are also critical for the development of LTi cells in the fetus in a dose dependent manner [45] and maternal high fat diet exposure can increase IL-17-producing ILC3 in the offspring intestine [4]. Although not specific to ILC3, there is an increasing evidence of microbial metabolites crossing from the mother into the fetal tissues and fetus to alter offspring development [46, 47].

It is noteworthy that germ free mice have defective ILC3 function, with specific decrease in IL-22-producing ILC3, demonstrating the importance of the microbiome for overall development [48]. There is an evidence that microbial induction of cytokines plays an important role in ILC3 activation. In particular, IL-1β and IL-23 production by myeloid cells can activate ILC3 as a result of microbial triggers [49, 50], mediated via MyD88 and Nod2 [51]. Human ILC3, unlike murine ILC3, express TLRs, and potentially may be directly functionally modulated by microbial colonization, which remains to be explored.

Dietary and microbial metabolites also modulate ILC3 function, as exemplified by short-chain fatty acids (SCFAs) and tryptophan metabolites. SCFAs are produced from bacterial fermentation of dietary fiber and include acetate, butyrate and propionate. Acetate and propionate supplementation in mice can increase IL-22 producing ILC3 in the colon and have a protective effect that is mediated by the SCFA receptor FFAR2 [52–54]. The type of SCFA is important, however, with butyrate having the opposite effect and decreasing the proportion of IL-22 producing ILC3 *in vitro* [55]. Tryptophan metabolites have also been found to be important for ILC3 function. Tryptophan is metabolized by bacteria (in particular *Lactobacilli spp*.) and produces ligands for AHR, including indoles [56]. Metabolites derived from bacterial strains belonging to Proteobacteria, Firmicutes, Fusobacteria and some Actinobacteria phyla can also activate AHR [37,57]. Several studies have demonstrated the importance of AHR signaling for ILC3 mediated development of isolated lymphoid follicles and cryptopatches postnatally [58–60]. Further, the absence of AHR ligands in the diet results in impaired IL-22 production by ILC3 in mice [61]. In human ILC3, the AHR agonist FICZ also stimulates ILC3 to produce IL-22 *in vitro*, supporting the critical role of AHR in maintaining ILC3 in humans as well [62]. There is the potential for maternal supplementation of these metabolites to enhance ILC3 function in offspring, but studies to date are absent.

### Studies in neonates implicating a critical role for ILC3 in disease pathogenesis

Newborns, and in particular, preterm infants, are susceptible to development of NEC, which has significant morbidity and mortality. The pathogenesis of NEC is believed to involve the interaction of an immature immune response with dysbiosis. The role of ILC3 in NEC pathogenesis has recently been examined in both murine and human studies. Cho *et al.* [3], examined the role of murine ILC3 in NEC pathogenesis and found in their model that NKp46^−^ ILC3 are increased. They further show that expression of intestinal tissue samples from humans with necrotic NEC had increased IL-17RA (IL-17A receptor) and decreased IL-22, compared with healthy intestinal tissue from infants without NEC. They found no difference in expression of the cytokine IL-17A. The decrease in IL-22 and increase in NKp46^−^ ILC3 suggests an imbalance in the proportion of IL-17A and IL-22 ILC3 subtypes may be involved in NEC pathogenesis. Babu et al., in a murine study, demonstrated that offspring born to mothers on high fat diet were more susceptible to a model of NEC, mediated by an increase in NKp46^+^ ILC3, which produced more IL-17, suggesting functional heterogeneity of ILC3 during the neonatal period [4]. The balance of ILC3 subtypes in the neonatal intestine may also be important as transgenic mice with overexpression of IL-23 and subsequent expansion of ILC3 have increased intestinal inflammation and neonatal death [63]. Combined, these data suggest a role of ILC3 in NEC but must be taken with caution given that the Cho et al. study utilized immunohistochemistry of paraffin embedded tissues and murine models of NEC are not the best representation of human disease. Further studies involving fresh tissue from patients with NEC that includes the characterization of ILC3 and other immune cell types that have been implicated in NEC, including T-cells [64, 65], are needed.

ILC3 may also play an important role in susceptibility to late onset sepsis associated with disruption of the microbiome by antibiotics [5, 6]. Neonatal mice have increased susceptibility to sepsis after exposure to antibiotics, even transiently, resulting from a decrease in IL-17-producing ILC3. In addition, in a murine neonatal pneumonia model, IL-22-producing ILC3 were reduced in the lungs of offspring born to mothers on broad spectrum antibiotics from 5 days prior to delivery and offspring were also more susceptible to pneumonia induced by *S. pneumoniae* [7]. The decrease in IL-22-producing ILC3 was partially restored with reconstitution of offspring with normal bacterial colonization postnatally [7]. BPD is a chronic respiratory condition that occurs in preterm infants as a result of prematurity, oxygen exposure and mechanical ventilation. In human neonatal bronchoalveolar lavage fluid, ILC3 are reduced in infants with BPD compared to infants without BPD [8]. It is also worthy to note that humans with RORC mutations and impaired ILC3 function have chronic candida infection [66], and preterm infants have increased susceptibility to fungal sepsis, again suggesting that preterm infants may have impaired ILC3 function.

**Figure 2:**
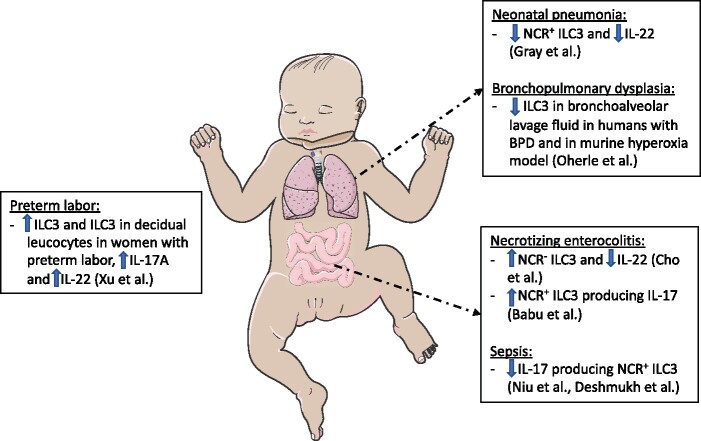
Summary of the implicated role of ILC3 in preterm and neonatal infants from published human and murine studies in pathogenesis of NEC, late onset sepsis, neonatal pneumonia, BPD and preterm labor

Recently, ILC have also been found in human placentas and associated with preterm labor. In a study that evaluated decidual leukocytes from placentas of women with preterm and term labor, the proportion of ILC2 and ILC3 was increased in women with preterm labor [2]. The authors also found that the expression of ILC3 cytokines IL-22 and IL-17A by flow was increased in women with spontaneous preterm labor. The results are intriguing and the source of the ILC3 (maternal versus fetal) and potential modulation by the maternal microbiome, intrauterine infection or altered metabolites remain to be explored. A summary of the changes in ILC3 subtypes extrapolated from the neonatal studies reviewed here is illustrated in Fig. 2.

### ILC3 in neonates: opportunities for future research

We are only just beginning to appreciate the importance of ILC3 in the pathogenesis of critical diseases involving the preterm and newborn infant. The functional heterogeneity of ILC3 in the newborn and preterm human infant needs to be further defined and mechanisms of control explored. Despite similarities in human and murine ILC3, there remain critical differences, with one of the most striking known to be the presence of TLRs on human ILC3. Also, the function of ILC3 subtypes in neonatal and adult models appears to differ. It is noteworthy that NCR^+^ ILC3 are conventionally thought to primarily produce the cytokine IL-22, but in neonatal mice this subtype appears to have a significant production of IL-17A associated with susceptibility to NEC and sepsis [4, 6]. The burden of NEC, BPD and sepsis on preterm infants is significant, and the potential for therapeutic interventions that target ILC3 function to improve outcomes is attractive. The mechanisms of how the maternal microbiome and diet modulate ILC3 in the fetus is another opportunity that could lead to therapeutic targets.

Future directions for research should include (i) analysis of human preterm and neonatal fresh intestinal tissue at baseline and in inflammatory states (e.g. NEC) to determine ILC3 ontogeny and define the subtypes of ILC3 present in this unique population, (ii) preclinical models that explore the fetal development of ILC3 subtypes with an emphasis on maternal environmental, microbial and dietary factors that impact lineage commitment and (iii) preclinical mechanistic studies that identify the pathways involved in ILC3 lineage commitment during the neonatal period, including further dissecting how local tissue factors and microbial colonization direct ILC3 heterogeneity and function. Given the growing evidence of the importance of ILC3 during the neonatal period, these studies have the potential of identifying unique microbial, nutrient and pharmaceutical therapies to maximize innate immunity in newborn infants by supplementing pregnant and lactating women or preterm infants.

## AUTHORS CONTRIBUTIONS

J.M. contributed to the writing, reviewing and editing of the original and final manuscript.

## FUNDING

This work was supported in part by the National Institutes of Health (NIDDK R01 DK121975).

## CONFLICT OF INTEREST STATEMENT

None declared.

## DATA AVAILABILITY STATEMENT

No new data were generated or analyzed in support of this work.
